# ﻿*Carexlinanensis* (sect. ﻿*Mitratae*), a new species of Cyperaceae from Zhejiang, East China

**DOI:** 10.3897/phytokeys.241.119176

**Published:** 2024-04-17

**Authors:** Xiang-Dong Qiu, Yi-Fei Lu, Xiao-Feng Jin

**Affiliations:** 1 School of Life Sciences, Gannan Normal University, Ganzhou, Jiangxi 341000, China Gannan Normal University Ganzhou China; 2 Zhejiang Provincial Key Laboratory of Forest Aromatic Plants-based Healthcare Functions and School of Forestry and Biotechnology, Zhejiang A&F University, Hangzhou, Zhejiang 311300, China Zhejiang A&F University Hangzhou China

**Keywords:** *Carexlinanensis*, East China, Mitrata clade, taxonomy, Zhejiang

## Abstract

*Carexlinanensis* X.D.Qiu & X.F.Jin, a new species in sect. Mitratae of the sedge family (Cyperaceae) from north-western Zhejiang is described and illustrated. We performed a statistical comparison of the new species with other closely-related species from the same section. *Carexlinanensis* is similar to *Carexsachalinensis* F.Schmidt, but differs in having leaf blades 1–2 mm wide (vs. 2.5–3.5 mm wide), utricles longer than pistillate glumes, with beak margin smooth (vs. barbate) and peduncles of lateral spikes enclosed in bract sheaths (vs. exserted from bract sheaths).

## ﻿Introduction

Cyperaceae, the sedge family with more than 5600 species, is the third largest monocot family and has considerable economic and ecological importance (Larridon et al. 2021). *Carex* L., the largest genus of 95 genera in the Cyperaceae, is distributed across all continents, except Antarctica (Larridon et al. 2021). This morphologically diverse genus contains > 2000 species and species thrive in various habitats, such as forests, meadows or thickets, wetlands or swamps, mountain slopes or sandy areas (Dai et al. 2010; Jin and Zhang 2020).

Kükenthal (1909) proposed an infrageneric classification of global *Carex* taxa, based on morphological characters, which has been challenged in recent phylogenetic studies (Jiménez-Mejías et al. 2016; Martín-Bravo et al. 2019; Roalson et al. 2021). Section Mitratae s.l. was established by Kükenthal’s worldwide monograph. This group was established to group species having nutlets frequently discoid-annulate at the apex and a persistent style base (Kükenthal 1909; Lu and Jin 2023). Recent phylogenetic studies have revealed that the sect. Mitratae s.l. is a polyphyletic group organised in five different clades within subg. Carex: Sect. Cryptostachyae and clades Tristachya, Truncatigluma, Mitrata and Conica (Roalson et al. 2021).

Recent ongoing field collections and specimen examinations of *Carex* in Zhejiang Province, China have resulted in the discovery and description of new species and subspecies (Lu and Jin 2018a), as well as some new distribution records (Lu and Jin 2018b; Lu et al. 2019; Zhang et al. 2020). These findings have contributed to revisions in the genus *Carex* for the new edition of Flora of Zhejiang (Jin et al. 2020; Jin et Lu 2021). Herein, we describe another new *Carex* species from north-western Zhejiang, which has nutlets discoid-annulate and thus belonging to sect. Mitratae s. l.

## ﻿Materials and methods

### ﻿Delimitation of study group

Specimens of the new species were collected from Mount Daming of north-western Zhejiang, China. A review of the related literature (Koyama 1962; Egorova 1999; Tang 2000; Hoshino et al. 2011) and a careful comparison of type specimens revealed that the new species is similar to those of the *Carexpisiformis* complex.

### ﻿Observation and analysis of macromorphological characters

Type specimens of the *Carexpisiformis* complex were critically examined including specimens at GH, K, KYO, LE, P, TI, WUK and ZJFC. Holotypes, syntypes or lectotypes of 15 species were measured and analysed.

A morphological exploration was conducted on eight diagnostic characters using PCoA (principal coordinate analysis) which was performed in RStudio software using ‘vegan’ and ‘ape’ packages.

Of these eight characters, five were binary (colour of basal sheaths: pale brown to brown vs. purple-brown; stolons: present vs. absent; indumentum on leaves and bracts: glabrous vs. pilose; lowermost bract: leaf-like vs. shortly leaf-like; and utricle beak: conspicuous vs. inconspicuous), one (leaf width) was continuous and two (colour of staminate spikes and indumentum on utricle bodies) were codified as ordered multistate using three or four categories (Table 1). According to the PCoA (see Results), the most morphologically similar species was the Japanese-Sakhalin endemic *Carexsachalinensis*. Accordingly, we performed subsequent statistical analyses focusing on comparing these two species. Twenty-four specimens from three known populations of the new species (holotype, isotypes and paratypes) and 12 specimens from Japan and Sakhalin of *C.sachalinensis* (see the note under Table 2) were measured. Based on our preliminary observations, we selected five characters: leaf width, length of staminate spikes, length of pistillate spikes, lowermost spike peduncle and utricles length.

**Table 1.** Comparison of diagnostic characters of the *Carexpisiformis* group.

Taxon	Voucher specimen	Colour of basal sheaths	Stolons	Leaf width (mm)	Indumentum on leaves and bracts	Lowermost bract	Colour of staminate spikes	Indumentum on utricle bodies	Utricle beak
*C.alterniflora*	*U. Faurie 453* (holo-P!)	pale brown to brown [0]	present [1]	2.25 ± 0.58	glabrous [0]	leaf-like, long than spike [1]	pale brown [1]	glabrous [0]	conspicuous [1]
*C.capilliformis*	*R.P. Farges* s.n. (holo-P!)	purple-brown [1]	absent [0]	0.72 ± 0.15	0	1	1	glabrous or nearly glabrous [1]	1
*C.clivorum*	*H. Yamamoto* s.n. (holo-KYO!)	0	0	2.43 ± 0.39	0	shortly leaf-like, shorter than spike [0]	white-green [0]	Pubescent [2]	1
*C.conicoides*	*K. Mayebara 155* (holo-KYO!)	0	1	2.30 ± 0.47	0	1	1	1	1
*C.duvaliana*	*Savatier* s.n. (holo-P!)	0	1	1.94 ± 0.49	pilose [1]	1	1	densely pubescent [3]	1
*C.fernaldiana*	*U. Faurie 4431* (holo-P!)	0	1	0.71 ± 0.17	0	1	1	0	1
*C.linanensis*	*Y. F. Lu & X. F. Jin 23042601* (holo-ZJFC!)	0	1	1.63 ± 0.40	0	0	1	1	inconspicuous [0]
*C.mayebarana*	*K. Mayebara* s.n. (holo. -P!)	0	1	2.25 ± 0.51	1	1	0	0	1
*C.pisiformis*	*Williams & Morrow 26* (synt-GH!)	0	1	1.94 ± 0.35	0	1	1	2	1
*C.polyschoena*	*U. Faurie 4928* (holo-P!)	1	0	1.96 ± 0.44	0	1	1	2	1
*C.polyschoenoides*	*G.G. Hsing 3728* (holo. -WUK!)	0	1	2.14 ± 0.36	0	0	1	1	1
*C.sachalinensis*	*Glehn* s.n. (lect-LE!)	0	1	3.22 ± 0.06	0	0	1	0	0
*C.sikokiana*	*Rein 3556* (holo. -P!)	1	1	2.93 ± 0.47	0	0	brown [2]	0	1
*C.stenostachys*	*Savatier 3370* (holo-P!)	0	0	2.19 ± 0.34	0	1	2	3	1
*C.tashiroana*	*Z. Tashiro* s.n. (holo-KYO!)	0	0	1.94 ± 0.30	0	1	0	1	0
*C.tenuinervis*	*K. Mayebara* s.n. (holo-KYO!)	0	0	1.84 ± 0.24	0	1	1	0	0

Note: the leaf width was measured and shown as Mean ± SD and the mean values were used for PCoA. The other values under the morphological characters were also used for PCoA, which have the same meaning in square brackets.

**Table 2.** The variation of five morphological characters between *C.sachalinensis* and the new species.

Characters	*C.linanensis*	*C.sachalinensis*	*p-value* (T test)
length of staminate spikes (mm) (mean ± sd)	13.45–39.50 (22.11 ± 5.22)	13.29–24.02 (17.76 ± 3.02)	< 0.01
length of pistillate spikes (mm) (mean ± sd)	7.91–20.11 (13.09 ± 2.65)	7.22–17.88 (11.39 ± 3.78)	> 0.05
utricle length (mm) (mean ± sd)	1.88–3.70 (2.80 ± 0.26)	2.69–3.28 (2.93 ± 0.18)	> 0.05
length of lowermost spike peduncles (mm) (mean ± sd)	5.18–31.01 (11.15 ± 4.45)	10.76–69.01 (37.70 ± 14.15)	< 0.01
leaf width (mm) (mean ± sd)	0.91–2.24 (1.63 ± 0.40)	1.29–4.34 (2.62 ± 0.59)	< 0.01

Note: Measured specimens of *C.sachalinensis* [Japan, Pref. Nagano, *M. Furuse 41247*, *28945* (NHN); Japan, Pref. Hakone, *M. Mizushima 912* (MICH, P); Japan, border between Pref. Miyagi and Pref. Yamagata, *A. Kimura s.n.* (MICH); Japan, Pref. Hakone, *P.A.L. Savatier 3496* (P); Japan, Pref. Tochigi, *P.A.L. Savatier 2223* (P); Japan, Mt. Fuji, *U.J. Faurie 1695* (P); Japan, Mt. Hakodate, *U.J. Faurie 582* (P); Japan, Pref. Tochigi, *U.J. Faurie 15559* (P); Russia, Sachalin, *F. Schmidt s.n.*, *241* (GH)].

### ﻿Observation on nutlet sculpture micromorphology

Scanning electron microscopy (SEM) observations of nutlet surface of the new species and the similar species *Carexsachalinensis* were carried out. Mature nutlets were gathered from the specimens ‘*Y. F. Lu & X. F. Jin 23042601*’ for the new species and ‘*O. Yano s. n.*’ for *C.sachalinensis*, respectively. The nutlets were initially soaked in a solution of concentrated sulphuric acid and acetic anhydride (volume ratio = 1:9) for 12 hours, then rinsed in acetic acid for 10 min and water for 5 min and placed in a bath-type ultrasonic cleaner for 7 min with 70% ethanol to remove the cuticle and outer periclinal wall of the epidermis (Jin et al. 2014). After air drying, the nutlets were mounted on stubs using double-sided adhesive tape and coated with a layer of gold. The coated utricles and nutlets were observed and photographed under a GEMINI-300 scanning electron microscope.

## ﻿Results

### ﻿Morphological study

**Figure 1.**
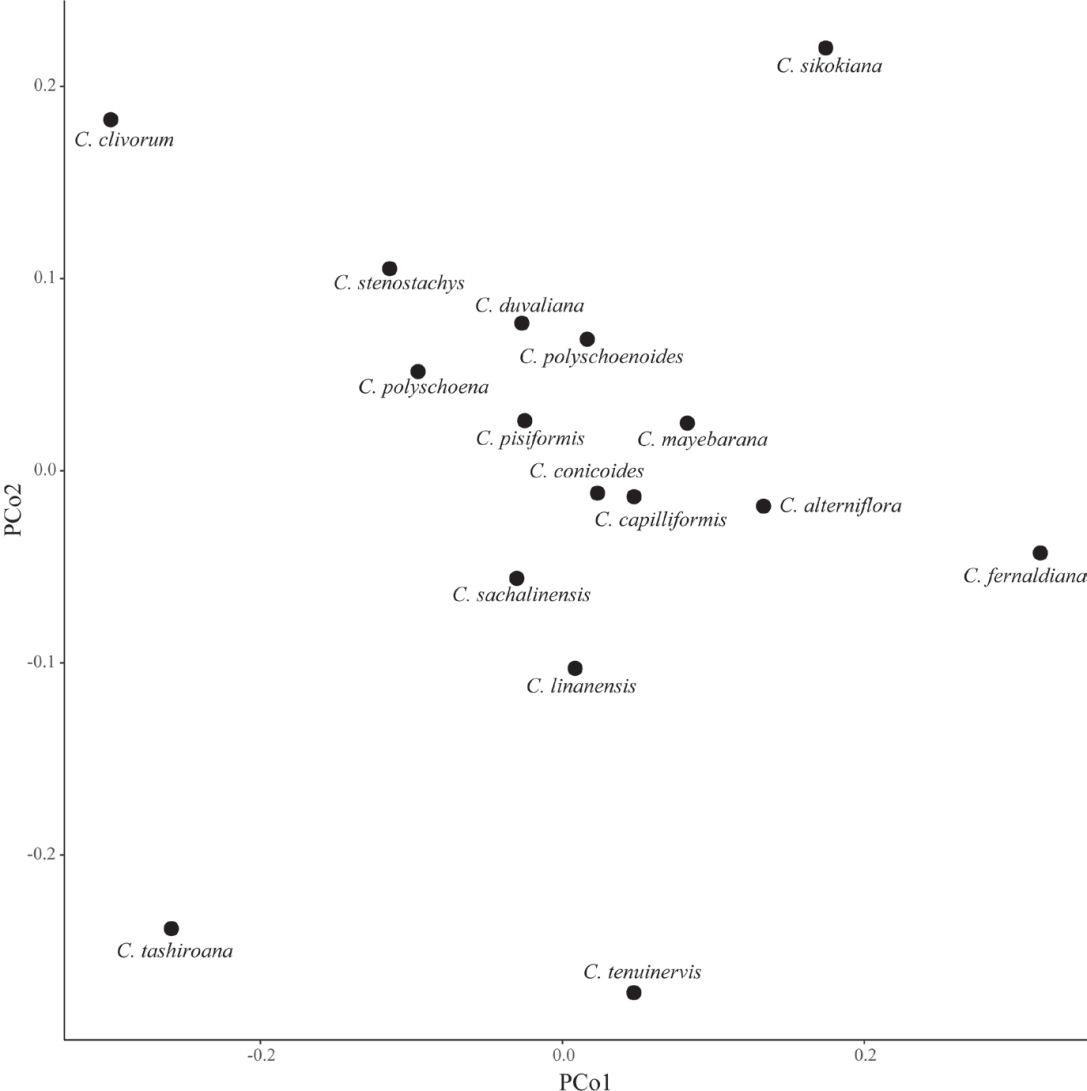
PCoA (principal coordinate analysis) of the *Carexpisiformis* group, based on analysis of eight diagnostic characters of the type specimens.

Based upon the PCoA of diagnostic morphological characters, the new species is most closely related to *C.sachalinensis* (Fig. 1), although it seems smaller than this in all its parts.

**Figure 2.**
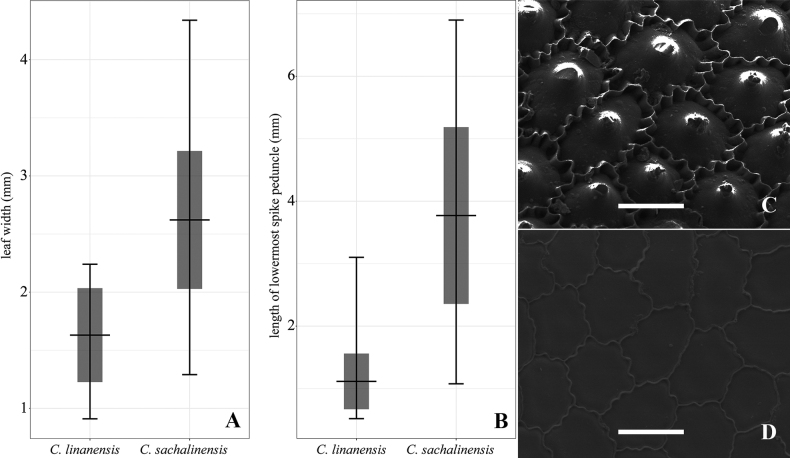
Differences between *Carexlinanensis* and *C.sachalinensis***A** variation of leaf width **B** variation of lowermost spike peduncle **C** surface sculpture of *C.linanensis***D** surface sculpture of *C.sachalinensis*. Scale bars: 20 μm.

The morphological variation of five characters, viz. leaf width, length of staminate spikes, length of pistillate spikes, lowermost spike peduncle and utricles length are shown in Table 2. T-test revealed significant differences in length of staminate spikes, length of lowermost spike peduncles and leaf width between the new species and *C.sachalinensis*. Amongst them, length of lowermost spike peduncles and leaf width were discontinuous between the new species and *C.sachalinensis* (Fig. 2A, B), while overlapping in length of staminate spikes was detected.

### ﻿Surface sculpture of nutlets

The micromorphology of nutlet surface sculpture of the new species and *C.sachalinensis* is shown in Fig. 2C, D. Both the new species and *C.sachalinensis* have the epidermal cells of nutlets 5–7-gonal, with undulated anticlinal walls. The silica platform of epidermal cells of the new species is strongly convex, with a solitary silica body, while that in the observed sample of *C.sachalinensis* is flat, without silica body.

### ﻿Taxonomic treatment

#### ﻿ Carex linanensis

Taxon classificationPlantaePoalesCyperaceae

X.D.Qiu & X.F.Jin sp. nov.

B2E905B7-72F4-5F5A-8650-42BE38A72A56

urn:lsid:ipni.org:names:77340286-1

Figs 3A–I , 4A –

##### Diagnostic description.

This new species is similar to *Carexsachalinensis* F.Schmidt in having lowermost bract shortly leaf-like and utricles short-beaked, but differs by having leaf blades 1–2 mm wide (vs. 2.5–3.5 mm wide), utricles longer than pistillate glumes, with beak margin smooth (vs. barbate) and lateral spikes 1 or 2 (vs. 2 or 3), with peduncles enclosed in bract sheaths (vs. enclosed in bract sheaths).

**Figure 3:**
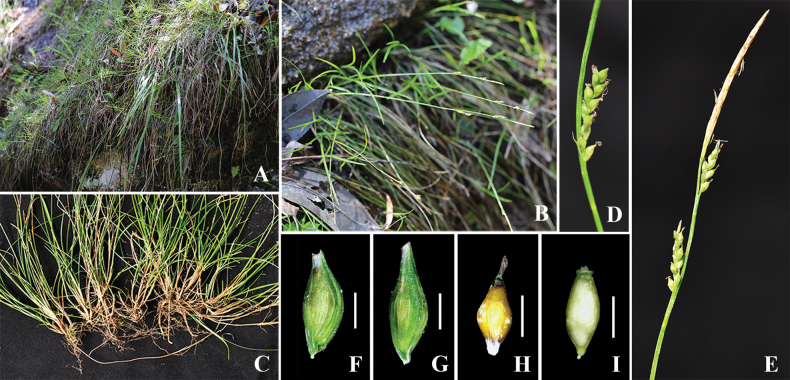
. *Carexlinanensis* sp. nov. **A, B** habitat **C** lower part of habit, showing stolons **D** pistillate spike and bract **E** staminate spike and two pistillate spikes **F, G** utricle **H, I** nutlet. Scale bars: 1 mm.

##### Type.

China. Zhejiang Province: Hangzhou City, Lin’an District, Changhua, Mount Daming, Pansidong, 30.033446°N, 118.987165°E, on cliffs along scenic boardwalk, 1460 m elev., 26 Apr 2023, *Y. F. Lu & X. F. Jin 23042601* (holotype: ZJFC!; isotypes: IBK!, ZJFC!, ZM!, PE!).

**Figure 4.**
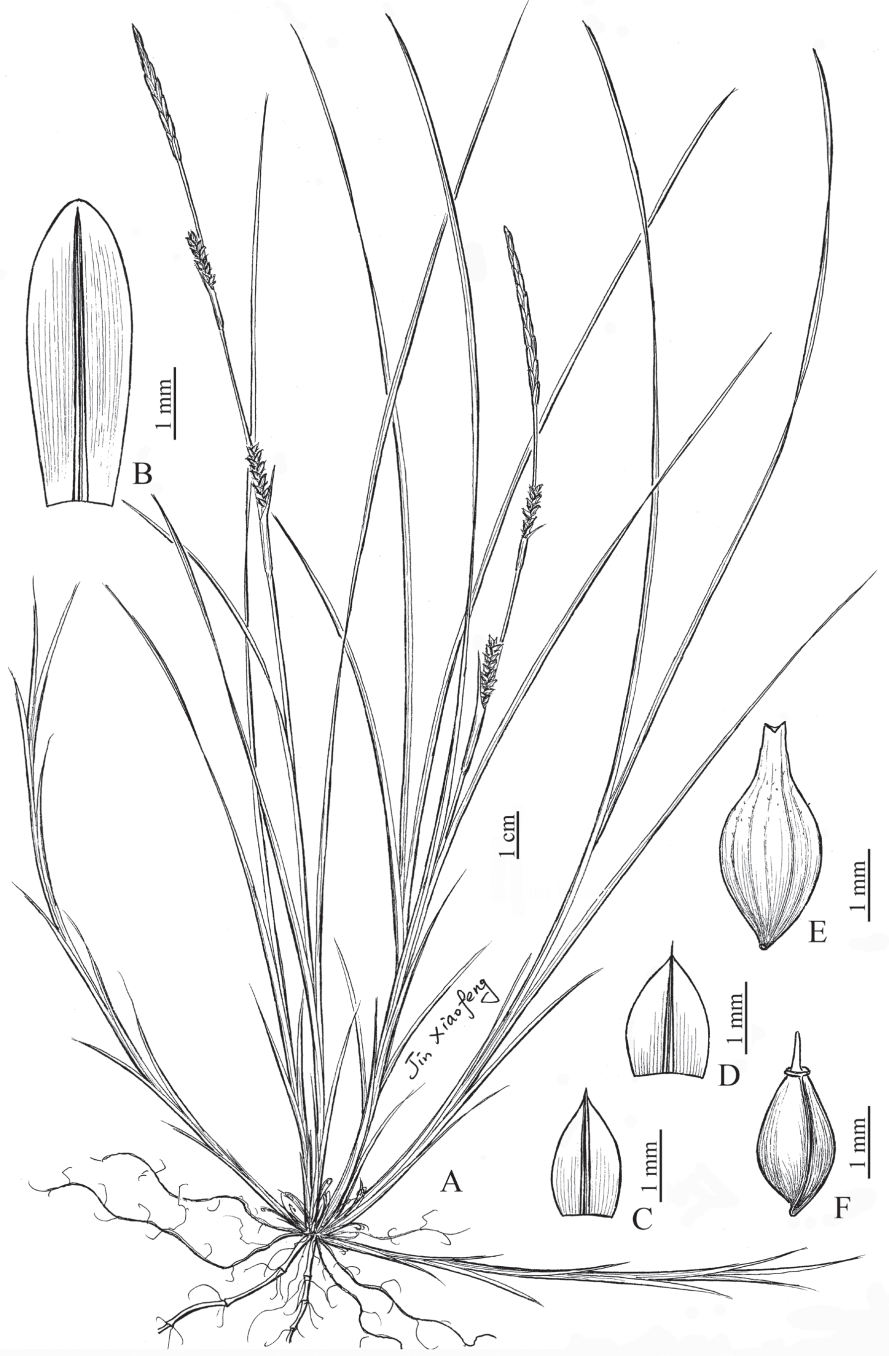
*Carexlinanensis* sp. nov. **A** habit **B** staminate glume **C, D** pistillate glume **E** utricle **F** nutlet (drawn by Xiao-Feng Jin; based on the holotype: *Y. F. Lu & X. F. Jin 23042601*).

##### Description.

Perennial herbs. Rhizomes obliquely ascending, slender, woody, stoloniferous. Culms 12–30 cm tall, obtusely trigonous, slender, slightly scabrous, glabrous, base with brown or pale brown sheaths, sometimes split into fibres. Leaves longer than culms or, sometimes, nearly equal in length; blades 1–2 mm wide, herbaceous, flat or margin slightly involuted, scabrous on upper margins and abaxial leaf surfaces. Lowermost bract shortly leaf-like, uppers setaceous, shorter than spikes, sheathed; sheaths 6–12 mm long, upper ones gradually shorter. Spikes 2 or 3; terminal spike staminate, clavate, 2–3.5(–4) cm long, 1.8–2 mm wide, long-pedunculate at base; lateral spikes pistillate, loosely flowered, cylindrical or shortly cylindrical, 6–15 mm long, 2–2.5 mm wide, base with peduncles enclosed in bract sheath. Staminate glumes oblong or oblong-elliptic, 4–4.5 mm long, pale yellow or pale brown, obtuse at apex, green or brown 3-veined dorsal costa. Pistillate glumes ovate or broad-ovate, 1.5–1.8 mm long, pale yellow-brown, acuminate at apex, green 3-veined dorsal costa excurrent into a mucro or short-awn. Utricles brown-green, obovoid-ellipsoid (excluding beak), obtusely trigonous, 2.8–3.2 mm long (including beak), longer than pistillate glumes, membranous, distinctly thinly veined, sparsely puberulent, base attenuate and stipitate, apex gradually contracted into a ca. 0.3 mm long beak, orifice 2-lobed with minute teeth. Nutlets tightly enveloped, ovoid, trigonous, pale brown or yellow-brown, ca. 2 mm long, slightly excavated below, base with a ca. 0.2 mm long stipe, apex abruptly discoid-annulate or inconspicuously discoid-annulate; style thickened at base; stigmas 3.

##### Etymology.

The specific epithet ‘linanensis’ refers to the type locality of this new species which was collected from Mount Daming of Lin’an District in Hangzhou.

##### Phenology.

Flowering and fruiting occur from late April to mid-May.

##### Additional specimens examined (paratypes).

China. Zhejiang Province: Hangzhou City, Lin’an District, Changhua, Mount Daming, Pansidong, 30.033446°N, 118.987165°E, on cliffs along scenic boardwalk, 1460 m elev., 26 Apr 2023, *Y. F. Lu & X. F. Jin 23042602* (ZJFC!, ZM!); the same locality, 30.033534N, 118.987040E, on cliffs, 1460 m elev., 2 May 2022, *Y. F. Lu & X. F. Jin 22050201* (IBK!, ZJFC!, ZM!), 14 May 2022, *Y. F. Lu 307* (ZJFC!, ZM!); Mount Daming, Jingmagang, on cliffs, 1050 m elev., 14 May 2022, *Y. F. Lu 306, 308* (ZJFC!, ZM!), the same locality, on cliffs, 1100 m elev., 14 May 2022, *Y. F. Lu 310* (IBK!, PE!, ZJFC, ZM!); Mount Daming, Fengshu Valley, on cliffs, 950 m elev., 14 May 2022, *Y. F. Lu 312* (IBK!, PE!, ZJFC, ZM!).

##### Conservation status.

Least Concern (LC). The new species is a common sedge and grows on cliffs or along the Fengshu Valley in Damingshan Scenic Park. All known populations are in well-protected areas. but are strongly influenced by tourist activity unrelated to this species, so that the species will need attention related to conservation (IUCN 2022).

##### Notes.

Tang (2000) recognised *Carexpisiformis* Boott as a high-polymorphic species which is distributed on the Islands of Sakhalin and Kurils, as well as in Japan and Korea to south-western and eastern China. *Carexsachalinensis*, *C.alterniflora*, *C.fernaldiana*, *C.polyschonea* and *C.conicoides* were synonymised, while *C.duvaliana*, *C.capilliformis* and *C.polyschoenoides* from China were treated as independent species. Koyama (1962) compared the *C.sachalinensis* group in morphological characters, chromosome numbers and distribution pattern, recognising only one species, *C.pisiformis*, with 15 varieties and several forms. Egorova (1999) considered *C.sachalinensis* to be a very polymorphic species, represented in Sakhalin and Kurils by the type variety. The other epithets for taxa distributed in Honshu and the southern islands of Japan were treated as different varieties. After a critical examination of type specimens, we suggest that the *C.sachalinensis* group is in need of revision, necessitating further study. We propose treating taxa within this group as independent species, with a detailed comparison of the diagnostic characters in Table 1.

Morphologically, the new species, *Carexlinanensis*, is similar to *C.sachalinensis* in having loosely tufted plants with stolons, bracts shortly leaf-like, shorter than spikes, utricles inconspicuously beaked, but differs by having narrower leaves (1–2 mm wide), utricles puberulent with beak margin smooth and peduncles of pistillate spikes enclosed in bract sheaths.

## Supplementary Material

XML Treatment for Carex linanensis

## ﻿Additional information

### Author ORCIDs

Yi-Fei Lu https://orcid.org/0000-0001-6095-3821

Xiao-Feng Jin https://orcid.org/0000-0002-8195-6738

### Data availability

All of the data that support the findings of this study are available in the main text.
